# Clinical and hemato-biochemical studies on fever of unknown origin in buffaloes

**DOI:** 10.14202/vetworld.2015.1225-1229

**Published:** 2015-10-23

**Authors:** Parmod Kumar, V. K. Jain, Ankit Kumar, Neelesh Sindhu, Tarun Kumar, Gaurav Charaya, Surbhi, Sandeep Kumar, Divya Agnihotri, Sridhar

**Affiliations:** 1 Department of Veterinary Medicine, Lala Lajpat Rai University of Veterinary & Animal Sciences, Hisar - 125 004, Haryana, India; 2 Teaching Veterinary Clinical Complex, Lala Lajpat Rai University of Veterinary & Animal Sciences, Hisar - 125 004, Haryana, India; 3 Department of Veterinary Physiology and Biochemistry, Lala Lajpat Rai University of Veterinary & Animal Sciences, Hisar - 125 004, Haryana, India

**Keywords:** biochemical parameters, buffaloes, clinical observation, fever of unknown origin, haematology

## Abstract

**Aim:**

The present study was undertaken to ascertain the clinical observation and haemato-biochemical studies on fever of unknown origin (FUO) in buffaloes which were presented for treatment at the Teaching Veterinary Clinical Complex (TVCC), Lala Lajpat Rai University of Veterinary and Animal Sciences (LUVAS), Hisar.

**Materials and Methods:**

The investigation was conducted on total 106 clinical cases presented at “TVCC, LUVAS, Hisar.” Diseased animals having history of fever and increased rectal temperature were considered for the current study. Diagnosis of FUO was done on the basis of negative parasitological examination, culture examination, fecal and urine test. The cases in which etiology could not be established (such as pneumonia, metritis, traumatic reticuloperitonitis, urinary tract infection, trypanosomosis, diaphragmatic hernia, Brucellosis, and foreign body) were considered as true cases of FUO.

**Results:**

Out of 106 clinical cases different etiologies were identified in 76 (71.70%) cases including pneumonia, traumatic pericarditis, trypanosomosis, bacteremia, etc. and 30 cases (28.30%) remained undiagnosed even after detailed investigation. The mean rectal temperature (104.43±0.16°F), respiration rate (56.57±1.51/min) and pulse rate (83.40±1.77/min) of animals (n=30) suffering from FUO were significantly higher, whereas ruminal movement (1.00±0.23) was significantly lower compared to healthy control group. The mean value of hemoglobin, lymphocytes, and packed cell volume were significantly lower, whereas mean value of neutrophils was significantly higher compared to that of healthy control animals. Mean value of serum levels of glucose, phosphorus, aspartate aminotransferase (AST), alanine aminotransferase (ALT), creatine phosphokinase (CPK), blood urea nitrogen (BUN), and creatinine were found to be significantly higher, whereas mean value of calcium value was significantly lower in all clinically affected animals compared to the healthy control group.

**Conclusion:**

About 28.30% cases of fever in buffaloes were found to be of unknown origin. Haemato-biochemical findings in cases of FUO in buffaloes revealed relative neutrophilia with lymphopenia, hyperglycemia, hypocalcemia, hyperphosphatemia, significantly increased AST, ALT, and CPK along with adversely altered kidney function indicators (elevated BUN and serum creatinine).

## Introduction

Fever of unknown origin (FUO) is defined as the temperature above 101°F on several occasions over a period of 3 weeks and failure to reach a diagnosis despite 1 week of investigations. Fever itself is not an ailment but sign of diseases having very vast and variable etiologies which includes abscesses, tuberculosis, urinary tract infections, endocarditis, hepatobilliary infections, osteomyelitis, rickettsia, chlamydia, systemic bacterial illnesses, parasitic infections, lymphomas, leukemias, solid tumors, autoimmune diseases, collagen vascular disease, malignant histiocytoma, regional enteritis, granulomatous hepatitis, drug-induced fever, inherited diseases, endocrine disorders, thrombophlebitis, polyarthritis, etc. [1].

FUO is characterized by prolonged, unexplained fever usually accompanied by sickness behavior, which consists of lethargy, depression, anorexia, sleepiness, hyperalgesia, and the inability to concentrate [2,3]. Fever is a major challenge for diagnosis in both human and veterinary medicine and requires logical and rational diagnostic plan as it may be caused by more than 200 diseases in humans [4]. In animals, it is accompanied by anorexia, wasting, depression, muscle weakness, decline in milk production causing heavy losses to the farmers.

Keeping in view, the significance and paucity of research work on FUO in buffaloes, this study was conducted in relation to diagnosis and haemato-biochemical changes in clinical cases suffering from FUO.

## Materials and Methods

### Ethical approval

In the present study, samples were collected from clinical cases. As per University rules for these samples approval of Institutional Animal Ethics Committee is not required.

### Place of study

The study was conducted in Department of Veterinary Medicine, College of Veterinary Sciences, Lala Lajpat Rai University of Veterinary and Animal Sciences (LUVAS), Hisar.

### Clinical examination and sample collection

The present study was conducted on total 106 clinical cases suffering from fever which were reported at Teaching Veterinary Clinical Complex (TVCC), LUVAS, Hisar. Ten apparently healthy buffaloes were also included in this study as control group.

### Differential diagnosis of cases suffering from fever

Diseased animals having history of fever and increased rectal temperature were considered for current study on FUO. These animals were investigated in detail for establishing diagnosis of underlying disease conditions. Haemato-biochemical parameters, radiography, blood culture, immunological examination, urine, and fecal examination were performed in all clinical cases. The cases in which etiology could not be established were considered as true cases of FUO.

### Anamnesis and clinical inspection

Detailed history of affected animals with regard to age, duration of fever, duration of anorexia and response to previous treatment was obtained from the animals owners and handlers. Complete clinical examination of the suspected animals which included rectal temperature, pulse rate, respiration rate, ruminal movements, examination of lymph node, and mucous membrane was done.

## Haematological examination

A 10 ml of blood sample was collected from each animal aseptically using ethylenediaminetetraacetic acid/heparin coated sterile vials from jugular vein of the affected as well as healthy control group animals for estimation of haematohaematological parameters like hemoglobin (Hb), packed cell volume (PCV), total erythrocyte count (TEC), total leucocyte count (TLC), differential leukocyte count (DLC), mean corpuscular volume (MCV), mean corpuscular hemoglobin (MCH), and mean corpuscular hemoglobin concentration (MCHC) by standard procedures [5].

### Serum biochemistry

A 10 ml of blood sample was collected from each animal aseptically using clot activator sterile vials from jugular vein of the affected as well as healthy control group animals for estimation of biochemical parameters including blood glucose, alanine aminotransferase (ALT), aspartate aminotransferase (AST), calcium (Ca), phosphorus (P), magnesium (Mg), blood urea nitrogen (BUN), creatinine, alkaline phosphatase (ALP), creatine phosphokinase (CPK), and total protein (TP) by standard procedure using commercial kits supplied by Trans Asia Biomedical Ltd.

### Parasitological examination

Blood smear were analyzed for blood hemoprotozoan parasites (Theileriosis, Babesiosis, and Anaplasmosis). Serum samples from the affected animals were also subjected to latex agglutination test for detecting the presence of *Trypanosoma evansi* antigen [6].

### Fecal and urine examination

Fecal samples were collected per-rectally was than processed for microscopic examination by floatation and sedimentation technique for nematode, trematode, and cestode eggs.

### Culture examination

Blood sample was collected in sterile vials aseptically. The samples were inoculated on 5% sheep blood agar (BA), Mac Conkey’s lactose agar (MLA) and Sabouraud dextrose agar plates with the help of a sterile 4 mm diameter platinum loop. The plates were incubated aerobically at 37°C for 24-48 h [7]. The cases in which diagnosis could not be established were considered as true cases of FUO.

### Statistical analysis

Data were analyzed by Independent Student’s t-test using SPSS computer software package.

## Results

Buffaloes (n=106) suffering from persistent/recurrent fever were brought for treatment to the TVCC, LUVAS, Hisar. After intensive diagnostic efforts, 76 cases of fever were confirmed for specific etiologies as shown in Table-1 and rest 30 cases (28.30%) were considered as true cases of FUO.

**Table-1:** Differential diagnosis of buffaloes (n=106) suffering from fever.

Diseases diagnosed	Number of cases (106)	Percentage
Pneumonia	18	16.98
Traumatic pericarditis	12	11.32
Positive for blood culture	12	11.32
Trypanosomosis	10	9.43
Peritonitis	9	8.49
Metritis	4	3.44
Diaphragmatic hernia	3	2.83
Impaction	2	1.88
Mastitis	2	1.88
Brucellosis	1	0.94
FMD (after recovery)	1	0.94
Urinary tract infection	1	0.94
*Balantidium coli* infection	1	0.94
FUO	30	28.30

FUO=Fever of unknown origin, FMD=Foot-and-mouth disease

### Culture examination

Out of all, 12 blood culture positive cases bacterial isolates were *Staphylococcus* spp. (four), *Escherichia coli* (four), *Streptococcus* spp. (two), *Pseudomonas* spp. (one), and *Corynebacterium* spp. (one).

### History and clinical examination

History about mean age, the mean duration of anorexia and fever, previous treatment given and response to treatment was also taken and measured statistically (Table-2). The mean rectal temperature (104.43±0.16°F), respiration rate (56.57±1.51/min), and pulse rate (83.40±1.77/min) of animals (n=30) suffering from FUO were significantly higher compared to healthy control group rectal temperature (101.38±0.24°F), respiration rate (19.10±0.96/min) and pulse rate (58.20±2.45/min). Ruminal movement (1.00±0.23) was significantly lower in all clinically ill buffaloes compared to healthy control group (2.96±0.26 per minute) as shown in Table-3.

**Table-2:** History of buffaloes suffering from FUO (mean±SE).

Mean±SE					

Age (years)	Duration of anorexia (days)	Duration of fever (days)	Mucous membrane (percent)	Lymph nodes (percent)	Response to previous treatment (percent)
5.40±0.30	16.30±2.67	25.43±1.52	Congested: 70 Pale: 13.33 Normal: 16.67	Swollen: 40 Normal: 60	No response: 80 Recurrence: 20

FUO=Fever of unknown origin, SE=Standard error

**Table-3:** Comparison between mean±SE of clinical vital parameters in healthy and buffaloes with FUO.

Clinical parameters	Healthy control group (n=10)	Buffaloes suffering from FUO (n=30)
Temperature (°F)	101.38±0.24	104.43±0.16**
Respiration (per min)	19.10±0.96	56.57±1.51**
Pulse (per min)	58.20±2.45	83.40±1.77**
Rumen motility (per two min)	2.96±0.26	1.00±0.23**

** Significant (p<0.01), FUO=Fever of unknown origin, SE=Standard error

### Haematological parameters

The blood values of Hb, TLC, DLC (Neutrophils, Lymphocytes, Eosinophils, Monocytes and Basophils), TEC, PCV, erythrocytic indices (MCH, MCV and MCHC) were determined in diseased and healthy control animals. The mean value of hemoglobin, lymphocytes and PCV were significantly lower whereas mean value of neutrophils were significantly higher compared to that of healthy control animals as shown in Table-4.

**Table-4:** Status of some haematological parameters in buffaloes with FUO and control animals.

Haematological parameter	Control (n=10)	Buffaloes suffering from FUO (n=30)
Hb (g/dL)	12.64±0.40	10.25±0.64*
TLC (10^3^/µL)	8.33±0.34	10.57±0.94
Neutrophils (%)	39.50±1.31	54.63±5.48*
Lymphocytes (%)	57.00±0.95	40.33±5.60*
Monocytes (%)	2.30±0.45	3.53±0.93
Eosinophils (%)	1.20±0.35	1.5±0.61
Basophils (%)	-	-
PCV (%)	36.90±1.21	30.08±1.31*
TEC (10^6^/µL)	5.84±0.13	5.15±0.26
MCV (fL)	63.18±1.44	58.83±2.83
MCH (pg)	21.64±0.37	20.11±1.22
MCHC (%)	34.25±0.78	34.09±2.11

* Significant (p<0.05), FUO=Fever of unknown origin, Hb=Hemoglobin, PCV=Packed cell volume, TEC=Total erythrocyte count, TLC=Total leucocyte count, DLC=Differential leucocyte count, MCV=Mean corpuscular volume, MCH=Mean corpuscular hemoglobin, MCHC=Mean corpuscular hemoglobin concentration

### Biochemical parameters

The blood values of AST, ALT, ALP, CPK, BUN, creatinine, glucose, total protein, calcium, phosphorus, and magnesium were determined in diseased and healthy control animals. Mean value of serum levels of glucose, phosphorus, AST, ALT, CPK, BUN, and creatinine was found to be significantly higher whereas mean value of calcium value was significantly lower in all clinically affected animals compared to the healthy control group as shown in Table-5.

**Table-5:** Status of some biochemical parameters in buffaloes with FUO and control animals.

Serum biochemical parameters	Control (n=10)	Buffaloes suffering from FUO (n=30)
AST (IU/L)	57.53±3.35	148.77±5.77**
ALT (IU/L)	33.89±2.05	240.82±30.63**
ALP (IU/L)	191.30±18.24	240.06±40.99
CPK (IU/L)	256.96±19.89	372.83±12.21*
BUN (mg/dL)	35.11±3.21	52.46±4.71*
Creatinine (mg/dL)	1.71±0.06	2.27±0.29*
Blood glucose (mg/dL)	68.73±2.89	116.32±15.57**
Total protein (g/dL)	8.13±0.37	7.16±0.40
Calcium (mg/dL)	9.88±0.21	7.28±0.37
Phosphorus (mg/dL)	4.93±0.19	6.03±0.48*
Magnesium (mg/dL)	2.51±0.11	2.55±0.26

* Significant (p<0.05),

** Significant (p<0.01), ALT=Alanine aminotransferase, AST=Aspartate aminotransferase, ALP=Alkaline phosphatase, CPK=Creatine phosphokinase, BUN=Blood urea nitrogen

## Discussions

Buffaloes (n=106) suffering from fever for a long period were investigated and out of this, in 30 animals (28.30%) diagnosis could not be established. These cases were considered as true cases of FUO. Many other authors [8-11] also reported similar findings. In current study, the most common cause of fever were respiratory infections (16.98%), followed by traumatic pericarditis (11.32%), positive for bacteria on blood culture (11.32%), trypanosomosis (9.43%), peritonitis (8.49%), metritis (3.44%), diaphragmatic hernia (2.83%), impaction (1.88%), mastitis (1.88%), brucellosis (0.94%), foot-and-mouth disease (panter syndrome) (0.94%), UTI (0.94%), and *Balantidium coli* infection (0.94%).

In all animals suffering from FUO, temperature of clinically ill buffaloes was significantly higher (104.43±0.16°F) as compared to healthy control group (101.38±0.24°F), which was in confirmation with the reports of two studies [12,13]. In current study respiration rate (56.57±1.51) and pulse rate (83.40±1.77) was significantly higher in all clinically affected buffalo compared to healthy control group which coincides with the finding of Dixit *et al.*, [13] and Mohamed *et al.*, [14]. Rumen motility was significantly lower (1.00±0.23/2 min) in the clinically affected animals compared to that of the control group. On examining mucous membrane were found congested, pale and normal in 70%, 13.33%, and 16.67% cases, respectively. Radostits *et al.*, [15] mentioned similar observation in cattle suffering from fever (41.6°C) and found that most of the animals were having congested mucous membrane. Lymph nodes were examined and found swollen in 40% cases but normal in the rest of animals (60%).

Haematological values of buffaloes suffering from FUO in the present study exhibited significantly lower hemoglobin and PCV. No significant variation in monocyte and eosinophil count was observed. Lower hemoglobin, haematocrit values in diseased buffaloes were probably as a result of anorexia due to prolonged fever and leading to poor body condition. Total leukocyte count was found to be non-significantly higher in diseased animals compared to that of animals of control group which may be due to some obscure disease. Erythrocytic indices (MCV, MCH, and MCHC) were non-significant compared to healthy control animals which were found to be in agreement with Konigsson *et al.*, [16].

Serum levels of AST, ALT, and CPK were significantly higher in all clinically affected animals which were in agreement with the findings of few researchers [17,18].

Serum values of BUN and creatinine were found to be significantly increased in all clinical cases suffering from FUO compared to control group. Similar observations were made by several workers [14,19,20]. Any of the body processes inducing protein catabolism like anorexia can lead to increase in BUN.

The serum glucose level was significantly higher in affected animals. Alici *et al*. [21] concluded that plasma ACTH and serum cortisol elevations are common in infectious diseases, and they are more sensitive to increasing of body temperature and this increase in cortisol level during febrile period leads to hyperglycaemia. So, disturbance in this mechanism can lead to hyperglycemia during febrile period [22]. Hypocalcemia was observed during fever which can be attributed to requirement of large number of calcium ions for stimulation of insulin secretion in response to glucose. Remarkable fall in serum calcium level and rise in phosphorus levels was recorded in clinical cases of FUO in buffaloes compared to that of control animals which was in accordance with findings of Kaneko *et al.*, [22] and Hasanpour *et al.*, [23]. Hyperphostaemia further could be responsible for the secondary decrease in calcium level because of the Ca and P homeostasis in ruminants [24]. Kaneko *et al*. [22] also mentioned that hyperphophatemia leads to reciprocal reduction in serum ionized calcium because of mass law interactions between phosphate and calcium ions and because of decrease in renal 1, 25-dihydroxycholecalciferol synthesis by the kidneys.

## Conclusion

About 28.30% cases of fever in buffaloes were found to be of unknown origin. Haemato-biochemical findings in cases of FUO in buffaloes revealed relative neutrophilia with lymphopenia, hyperglycemia, hypocalcemia, hyperphosphatemia, significantly increased AST, ALT, and CPK along with adversely altered kidney function indicators (elevated BUN and serum creatinine).

## Authors’ Contributions

PK, VK, and SR proposed the study. AK, GC, and PK carried out the sample collection and diagnostic part. SK, AK, and PK done the biochemical analysis part. NS and DA analyzed the blood samples for hematological studies. NS, TK, AK, and PK critically observed the data and calculated mean using SPSS statistical software. PK, GC, and S prepared the manuscript. Finalization of manuscript was done by VK, SR, TK, and NS. All authors read and approved the final manuscript.
